# Gender affirming hormone therapy dosing behaviors among transgender and nonbinary adults

**DOI:** 10.1057/s41599-022-01291-5

**Published:** 2022-09-07

**Authors:** Arjee Restar, E. J. Dusic, Henri Garrison-Desany, Elle Lett, Avery Everhart, Kellan E. Baker, Ayden I. Scheim, S. Wilson Beckham, Sari Reisner, Adam J. Rose, Matthew J. Mimiaga, Asa Radix, Don Operario, Jaclyn M.W. Hughto

**Affiliations:** 1 Department of Epidemiology, School of Public Health, University of Washington, Seattle, WA, USA.; 2 Department of Behavioral and Social Sciences, Yale University School of Public Health, New Haven, CT, USA.; 3 Center for Applied Transgender Studies, Chicago, IL, USA.; 4 Department of Biostatistics, School of Public Health, University of Washington, Seattle, WA, USA.; 5 Department of Health Policy and Management, Johns Hopkins Bloomberg School of Public Health, Baltimore, MD, USA.; 6 Perelman School of Medicine, University of Pennsylvania, Philadelphia, PA, USA.; 7 Population, Health, & Place Program, Spatial Sciences Institute, Dornsife College of Letters, Arts, & Sciences, University of Southern California, Los Angeles, CA, USA.; 8 Whitman-Walker Institute, Washington, DC, USA.; 9 Department of Epidemiology and Biostatistics, Dornsife School of Public Health, Drexel University, Philadelphia, PA, USA.; 10 Department of Health, Behavior and Society, Johns Hopkins Bloomberg School of Public Health, Baltimore, MD, USA.; 11 Department of Epidemiology, Harvard T.H. Chan School of Public Health, Harvard University, Boston, MA, USA.; 12 Hebrew University School of Public Health, Jerusalem, Israel.; 13 UCLA Center for LGBTQ+ Advocacy, Research & Health, Los Angeles, CA, USA.; 14 Department of Epidemiology, UCLA Fielding School of Public Health, Los Angeles, CA, USA.; 15 Department of Epidemiology, Columbia University, New York, NY, USA.; 16 Callen-Lorde Community Health Center, New York, NY, USA.; 17 Departments of Behavioral and Social Sciences and Epidemiology, Brown University School of Public Health, Providence, RI, USA.; 18 Fenway Health, The Fenway Institute, Boston, MA, USA.

## Abstract

Gender-affirming hormones have been shown to improve psychological functioning and quality of life among transgender and nonbinary (trans) people, yet, scant research exists regarding whether and why individuals take more or less hormones than prescribed. Drawing on survey data from 379 trans people who were prescribed hormones, we utilized multivariable logistic regression models to identify factors associated with hormone-dosing behaviors and content analysis to examine the reasons for dose modifications. Overall, 24% of trans individuals took more hormones than prescribed and 57% took less. Taking more hormones than prescribed was significantly associated with having the same provider for primary and gender-affirming care and gender-based discrimination. Income and insurance coverage barriers were significantly associated with taking less hormones than prescribed. Differences by gender identity were also observed. Addressing barriers to hormone access and cost could help to ensure safe hormone-dosing behaviors and the achievement trans people’s gender-affirmation goals.

## Introduction

Access to gender-affirming hormones is crucial to many transgender and nonbinary (trans) individuals’ mental health and well-being. While not all trans individuals will seek out hormones, access to and use of hormones can be lifechanging among those who do, particularly those with gender dysphoria. Individuals with gender dysphoria experience distress related to differences between their sex assigned at birth and gender identity (Ashley, 2021) and may experience episodes of distress, ruminative thinking, anxiety, and depression (Bouman et al., 2017; Chodzen et al., 2019; Klemmer et al., 2021; Silva et al., 2021). As gender-affirming hormones are highly effective in developing secondary sex characteristics and are less costly and more accessible than gender-affirming surgeries, hormones are most often the first or only form of gender-affirming care trans patients will seek out (Restar et al., 2019; White Hughto & Reisner, 2016). Notably, hormone use has been shown to significantly improve psychological functioning and quality of life, reduce suicidal attempts and ideations, promote body satisfaction, and decrease gender dysphoria and is therefore considered medically necessary for many trans people (Bouman et al., 2017; Foster Skewis et al., 2021; Herman et al., 2019; Klemmer et al., 2021; White Hughto & Reisner, 2016).

Notably, there are several major barriers to accessing hormones and other forms of gender-affirming care, including systemic issues such as lack of insurance coverage, lack of availability of competent providers who prescribe hormones, and interpersonallevel experiences of bias and discrimination (James et al., 2021; Lerner et al., 2021; Puckett et al., 2018; Sperber et al., 2005). Many studies also find that trans people experience financial barriers to accessing hormones due in part to the fact that trans people are less likely than cisgender people to have health insurance (Lerner et al., 2021) and many insurance plans do not cover the cost of gender-affirming medical interventions (James et al., 2021; Lerner et al., 2021), despite the fact that gender-affirming care is a costeffective intervention (Baker, 2017). Discrimination and mistreatment in clinical encounters also present barriers to accessing hormones, such as providers asking invasive questions, refusing care, verbally harassing or using abusive language, and physically abusing trans patients (Hoffkling et al., 2017; Lerner et al., 2021; Redfern & Sinclair, 2014; Sperber et al., 2005). Even when providers do not explicitly discriminate or mistreat their trans patients, they often decline or refuse to provide adequate care for this population due to transphobia, lack of clinical and cultural competency, or both (Hughto et al., 2015; Lerner et al., 2021). Lack of education on how to care for and interact with trans patients creates negative interactions between patients and providers, which can lead to future avoidance of care and medical mistrust on the part of trans people (Hughto et al., 2015; Johnson et al., 2020; Lerner et al., 2021).

The use of gender-affirming hormones in a manner that is inconsistent with prescribed dosages can have adverse clinical and health consequences (Webb et al., 2020). Research on general medication adherence has shown that many factors influence an individual’s decision and ability to follow a prescribed treatment plan. Indeed, economic barriers, convenience, and poor communication with prescribing physicians have been shown to influence whether an individual will take medication as prescribed (Ratanawongsa et al., 2013; Sabaté & Sabaté, 2003; Zolnierek & DiMatteo, 2009). However, to our research team’s knowledge, there are currently no studies that detail why some individuals do not take hormones as prescribed. To fill this gap, the primary objective of this exploratory study is to identify the sociodemographic, healthcare indicators, and discrimination experiences associated with taking more or less hormones than prescribed, as well as trans people’s reasons for modifying their prescribed dose.

## Methods

### Study sample and procedures.

This is a secondary cross-sectional analysis of survey data from Project VOICE (Voicing Our Individual and Community Experiences), a needs assessment led by the Fenway Institute at Fenway Health (Fenway) and the Massachusetts Transgender Political Coalition (MTPC). Between March and August 2019, trans residents of Massachusetts (MA) and Rhode Island (RI) were surveyed about their sociodemographics, healthcare experiences, and health. Respondents were purposively sampled and recruited via venues where trans people congregate, including online sites such as listservs and community-based social networking webpages, as well as inperson sites such as trans-specific community events and trans-friendly clinics. Participants were eligible for the study if they were 18 years or older, self-identified as transgender or non-binary, resided in MA or RI, were willing to provide electronic written informed consent, and spoke either English or Spanish. Eligible respondents who completed the survey were invited to opt into a community raffle for one of 54 gift cards, with values ranging from $10 to $250. Additional details on the study procedures can be found elsewhere (Restar et al., 2020).

The present analysis focuses on a subsample of 379 trans respondents who indicated that they were currently taking hormones as part of their gender-affirmation care. This secondary analysis aimed to identify characteristics of trans respondents who reported taking more hormones than prescribed and respondents who reported taking less hormones than prescribed and to descriptively detail reasons for these modifying their hormone-dosing behaviors.

### Measures

#### Sociodemographic.

Respondent’s age was assessed in years and recoded as young adult (age 18–29) vs. all others (age 30+). Race/ethnicity was asked as a check-all-that-apply question and combined into White (non-Hispanic) vs. People of Color (POC, including Asian/Pacific Islander, Black, Hispanic/Latino, another race, and multiple races/ethnicities). Following the best-practice two-step method to assess gender (Reisner et al., 2016), we combined two items on assigned sex at birth (female, male) and current gender identity to denote respondents who are transfeminine, transmasculine, or nonbinary (e.g., genderqueer, gender non-conforming). Respondents were then asked if they were currently employed for wages or not. Lastly, low income was recoded if personal income fell below $30,000 (vs. not).

#### Healthcare experiences and discrimination.

A series of questions about health insurance, routine care, and gender-affirming care were asked. First, health insurance coverage for hormones was recoded to “yes” if it was covered, vs. “no” if it was not covered or the patient had no insurance. Respondents were then asked if they go to the same provider for both primary and gender-affirming care, with response options as either “yes, I go to the same provider for both types of care” or “no, I see a different provider for each type of care.” Respondents were asked in years when they received routine care last, and responses were dichotomized as “yes” if care was received within the past year vs. “no.” Similarly, mental health treatment within the past year was dichotomized as yes/no.

A series of questions about past year routine care avoidance were asked. Respondents indicated “yes” if, within the past year, they have postponed or did not try to get check-ups or other preventative medical care because of (a) gender-based mistreatment, (b) not being able to afford care, or (c) a doctor or other provider refused to treat them.

To assess major gender-based discrimination experiences, respondents were asked whether, in the past year, they had experienced the following because of their gender identity: (a) discouraged by a teacher or advisor from seeking higher education, (b) denied a scholarship, (c) not hired for a job (d) not given a promotion, (e) fired, (f) prevented from renting or buying a home in the neighborhood you wanted, (g) prevented from remaining in a neighborhood because neighbors made life so uncomfortable, (h) hassled by the police, (i) denied a bank loan, (j) provided inferior service by a store/restaurant employee, plumber, mechanic, or service provider, (k) denied medical care. Responses were coded as “1” if yes or “0” if no and were summed to create a continuous score of major gender-based discrimination experiences (range: 0–11).

#### Hormone therapy dosing behaviors (outcome).

Hormone-dosing behaviors were assessed via two questions that asked respondents [1] how often they take more hormones than prescribed; and [2] how often they take less hormones than prescribed. The questionnaire provided the following definition: “taking what is prescribed means taking the right dose at the time as instructed by a healthcare provider.” Response options for both questions ranged from never to always and were recoded as yes (rarely, sometimes, most of the time, always) vs. no (never).

In a check-all-that-apply question design, respondents who reported taking more or less hormones than prescribed were asked to provide detailed information about why they took their hormones other than as prescribed. For those who indicated that they were taking more hormones than prescribed, potential reasons included the following: (a) I think that taking more hormones will speed up my transition/gender-affirmation process, (b) my friends suggested I should take more, (c) I don’t trust my doctor/healthcare provider’s advice, (d) I do not think my doctor/healthcare provider is giving me the right dose, (e) My hormones make me feel good, or (f) Other, please specify. Similarly, for those who indicated that they were taking less hormones than prescribed, potential reasons included the following: (a) I cannot afford it, (b) I have no health insurance, (c) I forget to take it, (d) I forget to pick up my prescription, (c) I get it through friends, online, or on the street, and it’s not always available, (d) I do not trust my doctor/healthcare provider’s advice, (e) I do not think my doctor/healthcare provider is giving me the right dose, (f) It is hard for me to get to my doctor’s appointment to get the prescription, (g) My doctor/healthcare provider said I didn’t need to take it, (f) I am afraid my hormones will not work well with the other medications I take, (g) I am worried about gaining weight, (h) My hormones make me feel sick, (i) I am not sure I want to take hormones anymore, (j) Other, please specify. Response options to these questions were based on feedback from community partners and medical providers as key informants involved in the survey design.

### Data analysis.

Univariate descriptive statistics [mean, standard deviation (SD), frequency, and proportions] were performed to examine the overall distribution of the final analytical sample, overall (*n* = 379) and stratified by the two hormone-dosing questions. We also used bivariate analyses to examine patterns of hormone-dosing behaviors based on respondents’ sociodemographic characteristics, healthcare experiences, and discrimination.

Next, we performed a multivariable analysis using logistic regression to assess relationships between the independent variables (i.e., sociodemographic, healthcare experiences, and discrimination) and our main outcome (i.e., took more hormones than prescribed, took less hormones than prescribed. We then constructed two separate multivariable regression models, one for each outcome. Given the exploratory nature of this study, prior to building our models, we utilized a lasso procedure to select key variables to include in the model (Tibshirani, 1996). Given our modest sample size, we used nonparametric bootstrapping with 1000 iterations to estimate confidence intervals and reduce Type 1 error per model (Parra‐Frutos, 2014). The significance level was set to *p* < 0.05 a priori. We used Stata-MP version 17.0 to perform all statistical analyses.

Finally, we calculated the frequency of each reason for taking more or less hormones. We then utilized content analysis to examine the write-in responses under the category “other” (Kohlbacher, 2006). Each emergent theme was descriptively analyzed and included in the final list of reasons.

### Ethics.

All enrolled respondents provided their electronic, written informed consent, which detailed the voluntary nature of their participation and their rights to confidentiality and privacy. All study activities were approved by the Fenway Institutional Review Board (IRB).

## Results

### Sample characteristics.

Sample characteristics are shown in Table 1. About half of the respondents were young adults under the age of 30 (51%). The majority of respondents were White non-Hispanic (82%). A third of participants (33%) were transfeminine, 44% transmasculine, and 23% nonbinary. More than half of the sample was employed for wages (70%), and more than half reported having a low income (53%).

The majority of the sample reported having health insurance that covers hormones (85%), having the same provider for primary and gender-affirming care (74%), and receiving routine care in the past year (83%). A third received mental health treatment in the past year (37%). A total of 19% reported avoiding routine care in the past year due to gender-based mistreatment, and 26% avoided care due to cost. A total of 6% reported experiencing having a provider who refused them treatment in the past year. The mean number of major gender-based discrimination experiences in the past year was 1.7 out of a possible 11 (standard deviation [SD] = 1.9).

Overall, 24% of the sample reported taking more hormones than prescribed, and 57% reported taking less hormones than prescribed at some point in their lives. Less than one-fifth (19%) did not report modifying their hormone dosage. Among those who took more hormones than prescribed (*n* = 90), 44.4% were transfeminine, 33.3% were transmasculine, and 22.2% were nonbinary respondents. Among those who took less hormones than prescribed (*n* = 215), 27% were transfeminine, 46% were transmasculine, and 27% were nonbinary respondents.

### Regression outcome: taking more hormones than prescribed.

Table 2 shows the adjusted multivariable logistic regression models examining factors associated with taking more hormones than prescribed. In the final model, the odds of taking more hormones were lower among respondents who identified as transmasculine compared to transfeminine (adjusted OR [aOR]=0.45, 95% confidence interval [95% CI] = 0.23–0.88) and among low-income respondents (aOR = 0.41, 95% CI = 0.20–0.82). The odds of taking more hormones than prescribed were higher among respondents who reported having the same provider for primary and gender-affirming care (aOR = 2.14, 95% CI = 1.04–4.44) and those with an increased number of major gender-based discrimination experiences out of a possible 11 (aOR = 1.25 per experience, 95% CI = 1.08–1.44).

### Regression outcome: taking less hormones than prescribed.

Table 2 also shows the adjusted multivariable logistic regression models examining factors associated with taking less hormones than prescribed. In the final model, the odds of taking less hormones than prescribed were higher among nonbinary respondents compared to transfeminine respondents (aOR = 2.06, 95% CI = 1.05–4.04), those with a low income (aOR = 1.94, 95% CI = 1.13–3.32), those with no insurance coverage for hormones (aOR = 4.27, 95% CI = 1.73–10.56), and those who had received mental health treatment in the past year (aOR = 2.00, 95% CI = 1.14–3.48).

### Reasons for taking more hormones.

As shown in Table 3, the most endorsed reasons for taking more hormones than prescribed were believing that it is not the right prescribed dose (37%), taking it to feel good (36%), to speed up transition or the gender-affirmation process (27%), and making up for missed doses (17%).

Emergent themes from the write-in responses included the following reasons for taking more hormones: making up for missed doses, having concerns about reproductive health, and having an imprecise practice of dose administration.

Many respondents reported making up for missed hormone doses as a reason for taking more hormones than prescribed, with some respondents indicating “doubling” or taking “a little extra” dose if missed. These respondents noted:

“If I miss a week because my pharmacy took forever to get my T (testosterone) in, I’ll sometimes go to 0.45 on the 1 mL syringe instead of 0.4.”

(transmasculine respondent, age 24)

“Accidentally doubling my dose because I forgot that I took the first.”

(transfeminine respondent, age 37)

“I miss my shot day; I accidentally pull a little extra when getting the shot ready.”

(nonbinary respondent, age 29)

Reproductive health concerns regarding menstruation also emerged as one of the reasons transmasculine people reported taking more hormones than prescribed. Specifically, many transmasculine respondents reported taking higher doses of their hormones to mitigate one’s gender dysphoria and physical discomfort related to menstruation. Participants noted:

“Experience menstrual cramping.”

(transmasculine respondent, age 45)

“If I start bleeding.”

(transmasculine respondent, age 29)

“Period returned making me feel dysphoric.”

(transmasculine respondent, age 28).

Lastly, some respondents noted that taking more hormones than prescribed also occurred when they experienced logistical difficulty self-administering hormones via injection, as preparing the dose can sometimes lead to “imprecise” measurement. Specifically, respondents mentioned that they often increase their dose as it is “easier to go over than try to be exact” or compensate for when the medication “leaks” out of the syringe, as described below:

“I take a bit more most times because some usually leaks out.”

(transmasculine respondent, age 19).

“Measurement is imprecise and I honestly don’t care about getting it perfect—easier to go over than try to be exact.”

(transmasculine respondent, age 37)

### Reasons for taking less hormones.

As shown in Table 3, the most commonly endorsed reasons for taking less hormones than prescribed were forgetting to take the medication (70%), forgetting to pick up the prescription (27%), cost of hormones (18%), experiencing transportation barriers when attempting to pick up their prescription (13%), having syringe concerns (e.g., phobia, pain, anxiety) (9%), health insurance issues (e.g., lack of insurance, delay in approval, changes in in-network provider) (7%), and believing that the prescribed dose was incorrect (5%).

Among those who indicated taking less hormones than prescribed, emergent themes included experiencing other physical concerns (e.g., hair loss, acne issues), pharmacy issues (e.g., delays or unavailability of refills, being stigmatized by the pharmacist), and reliance on other people to administer their hormones.

Some participants who endorsed taking less hormones than prescribed reported experiencing psychological and physical concerns about using syringes, particularly pain at the injection site, as well as anxiety and phobia related to the injection. These negative experiences and concerns with syringes could deter some participants from successfully taking their medication, as described by the following respondents:

“Intense anxiety about injecting prevents me from completing a shot.”

(nonbinary age 20)

“Injection method is uncomfortable.”

(transmasculine, age 26)

“Injection site pain/fatigue.”

(transmasculine, age 32)

“The injections are painful, so I often procrastinate on it.”

(transmasculine, age 39)

Moreover, experiencing pain from injecting could also delay the timing of hormone administration. For instance, one respondent who reported taking less hormones than prescribed elaborated on how they would adjust the frequency of taking their hormones by 5 or more days to mitigate injection pain. They noted:

“I use IM (intramuscular) injections for hormones; it is painful to use needles, so rather than every 7 days as prescribed, I do once every 12 days.”

(transmasculine, age 33)

Another reason some respondents endorsed taking less hormones than prescribed is to mitigate the physical side effects of hormones. For instance, one nonbinary respondent described experiencing hair loss, and one transmasculine respondent reported experiencing acne, which would, in turn, led them to reduce their hormone dose to mitigate these side effects, as described below:

“It causes lots of hair loss so I just dab it on to feel good, but the regular application makes my hair fall out more.”

(nonbinary, age 36)

“To reduce acne issues.”

(transmasculine, age 29)

Additionally, one nonbinary respondent, while “liking all other effects” of testosterone, described developing distress associated with body hair grow and lowered their dose to minimize these physical changes:

“Dysphoria from body hair growth caused by T (testosterone), despite liking all the other effects.”

(nonbinary, age 29)

Some respondents described prescription fill-related barriers as a reason for taking less hormones than prescribed. These barriers ranged from prescription unavailability and refill delays to forgetting to call in refills, which made some respondents ration their hormones. As the following three respondents expressed:

“Pharmacy has hard time acquiring medication.”

(transmasculine, age 43)

“I’m afraid of running out/losing access to hormones and want to have a backup supply, or sometimes I forget to call in refills in time and have to stretch what I have left so I’m not off hormones cold turkey while I wait to get more.”

(transfeminine, age 26)

“Very afraid of running out and not being able to get more. It’s all I have left.”

(transfeminine, age 38)

Additionally, some participants reported experiencing gender-based discrimination by pharmacists when picking up their hormone prescriptions, which discouraged them from coming into the pharmacy again or caused them to delay or not obtain their hormones. This was noted by the following two respondents:

“It’s a restricted substance and the pharmacy always gives me grief trying to pick it up. It’s the only prescription I have [in which] pharmacists are weird with me about or call up my doctor for, and I’ve never had that happen even with other controlled substances.”

(transmasculine, age 19)

“Discrimination faced at pharmacies filling orders.”

(transmasculine, age 23)

Lastly, one respondent noted that they take less hormones than prescribed due to having to rely on others to administer their dose. This respondent noted,

I can’t administer it myself and have to rely on others.

(transmasculine, age 24)

## Discussion

To our knowledge, this is the first study to descriptively explore and detail dosing behaviors of prescribed hormones among trans populations. Taken together, our results indicate that access barriers related to income and insurance coverage were associated with trans respondents taking less hormones than prescribed whereas taking more hormones than prescribed was associated with having one’s primary care physician also prescribe hormone treatment, as well as with experiences of gender-based discrimination. While exploratory, these findings show a critical need for examining ways to optimize adherence to gender-affirming hormones by addressing multiple levels of individual and structural barriers that can deter trans people from meeting their gender-affirmation goals.

We found that participants were more likely to take less hormones than prescribed due to a range of factors reflecting both structural barriers to access and perceived incentives to take a reduced dose. Insurance issues were a major contributor to variable dosing: specifically, a lack of insurance coverage, whether due to being uninsured or having health insurance that did not cover hormones, was highly associated with taking a reduced dose, potentially due to rationing or the inability to consistently afford the cost of their hormone prescription with no insurance coverage. A prior study found that uninsured trans people were less likely to be on any hormone treatment than insured trans people (Stroumsa et al., 2020). Even for people with insurance coverage, exclusions of gender-affirming care, including hormone therapy, persist among people with public insurance (particularly state Medicaid programs) and people with private insurance plans (Dowshen et al., 2019; Kirkland et al., 2021; Zaliznyak et al., 2021). Similarly, while the literature on distance traveled to access gender-affirming services like hormones is scant, existing research illustrates a willingness to travel further distances to access knowledgeable providers that are capable of providing gender-affirming healthcare (Cicero et al., 2019; Kattari et al., 2020). While online prescribers are growing and expanding access to more areas, state-level policies on insurance, combined with controlled substance regulations, continue to vary state-by-state; thus access to online prescribers of gender-affirming hormones may be limited for trans people in certain geographic areas (Baker, 2017; Beauchamp, 2013; Holt et al., 2019; Kattari et al., 2020). Our findings related to insurance coverage, regulations, and provider availability suggest that, while some trans people in our study are able to access hormones, they may not be able to take the prescribed dose consistently. Cost issues may be driving hormone access issues as having a low-income was associated with taking hormones at lower doses than prescribed. Even with insurance coverage, individuals who have lower incomes may still be unable to consistently afford the co-pay for their hormone prescription and so they may reduce their hormone dosage as a way to ration their medication between refill cycles. These findings are important given previous research highlighting that trans people, despite having on average higher educational attainment than cisgender people, tend to have reduced employment and reduced income compared to the general population (Adams & Vincent, 2019; Seelman et al., 2017). To improve access to hormone treatment, both the availability and quality of insurance coverage, including overall insurance and medication affordability and specific provisions for the coverage of gender-affirming hormone therapy, should be improved in states across the country.

Receiving mental health treatment was also associated with reduced hormone use, though it is not clear whether this reflects a causal relationship or merely a co-occurring phenomenon. For instance, individuals receiving mental health treatment may do so because they struggle with daily functioning, including the functioning needed to receive and take hormones consistently. The challenge of taking hormones consistently is somewhat supported by the fact that forgetting to take hormones was the most commonly reported reason for missing a hormone dose. Prior studies have shown that non-attendance and non-adherence to physical health visits and medication are associated with poor mental health (Kretchy et al., 2014; Marrero et al., 2020). Receiving hormones has been associated with better mental health outcomes among trans people, including reduced depression and anxiety and improved quality of life (Baker et al., 2021; White Hughto & Reisner, 2016). However, other barriers and burdens may be associated with more severe mental health disorders that require treatment that may impact hormone use. Regardless of potential confounding, there should also be greater provider education about the particular barriers and gender-affirmation goals of nonbinary people seeking hormones.

We also found several associations with taking more hormones than prescribed. In particular, having the same healthcare provider for primary and gender-affirming care was associated with individuals taking doses beyond what was prescribed. From the write-in responses, overdosing and underdosing were both reported among those who mistrust their provider. In previous studies of trans people’s experiences in primary healthcare, one study found that 53.6% of trans participants reported that their primary care provider did not know enough about trans people to provide adequate care (Heng et al., 2018). Additionally, multiple studies reported that trans patients were educating their providers and conducting their own research (Costa et al., 2018; Dewey, 2008; Roller et al., 2015). Therefore, respondents may perceive that a primary care physician lacks the specialization to make informed dosage decisions and take dosing into their own hands. Our findings underscore the need for better integration of gender-affirming care with primary care, and vice versa, to optimize hormone adherence. Given differences between trans-friendly and trans-specific modes of service design and provision (Everhart et al., 2022), perspectives from trans patients regarding the ideal integration models, such as providing gender-affirming care in primary care settings vs. having primary care services in gender-affirming specialty clinics, could be helpful in understanding which models would feel more trustworthy, affirming, and likely to improve hormone adherence.

Individuals with greater reported experiences of gender-based discrimination were more likely to increase their dose beyond what was prescribed. This mechanism may be due in part to the desire to align one’s internal perceptions of one’s gender with one’s gender-affirming hormone goals. For example, individuals who are more likely to be perceived as nonbinary may experience increased gender-related discrimination (Anderson et al., 2020; Anderson, 2020; Cruz, 2014; Mizock et al., 2017), and therefore may feel compelled to take higher doses of hormones to achieve a more binary gender presentation and avoid discrimination. Future research should examine the reasons why individuals take higher hormone doses than prescribed, including the specific roles that discrimination and gender dysphoria play in trans people’s decisions around increased hormone dosing, particularly given that some trans people may not want to conform to binary gender expectations. It is also paramount that stigma and transphobia be addressed to reduce discrimination and support trans people’s health and well-being.

There were also gender-based differences in respondents reporting of taking more or less hormones than prescribed. Indeed, transmasculine respondents had reduced odds of taking more hormones than prescribed compared to transfeminine respondents. There are several potential mechanisms that may explain these findings, including that testosterone is highly regulated as a controlled substance, adding more structural barriers for providers, pharmacists, and trans people. Additionally, this finding might also be due to the prominence of secondary sex characteristics and perceived negative side effects of taking more testosterone compared to estrogen. For transfeminine people who begin hormones post-puberty, secondary sex characteristics may be perceived as more pronounced. Therefore, there may be a greater need to take increased doses to see intended results compared to transmasculine people, who may be likely to see quicker changes in their secondary sex characteristics at the prescribed dose. Additionally, while both estrogen and testosterone may yield unwanted side effects (Getahun et al., 2018; T’Sjoen et al., 2019), testosterone may be associated with a greater likelihood of experiencing unwanted effects, such as increased acne (Motosko et al., 2019), concerns around mood and aggression (Kristensen et al., 2021), and concerns that higher levels of testosterone will convert to estrogen and not produce the desired treatment effect. Although many of these concerns have been documented among people on testosterone there is a lack of empirical evidence to support many of these claims. For example, one large study found no association between serum testosterone levels and acne prevalence among transmasculine people, though age at the start of hormones was a risk factor for acne (Thoreson et al., 2021). Other studies have shown mixed evidence of changes in anger following testosterone therapy, with none following up longer than the first years on treatment (Defreyne et al., 2019; Kristensen et al., 2021; Motta et al., 2018; Thoreson et al., 2021). Further, while estradiol conversion is largely understudied among transmasculine participants, one small study from Massachusetts found estradiol levels decreased with testosterone treatment, with no evidence of conversion to estrogen greater than that in cisgender men (Chan et al., 2018). Nonetheless, concerns around unwanted side effects persist among some trans patients on hormone therapy, likely reflecting individual clinical histories and personal and community perceptions, rather than large-scale negative side effects across the population.

We also found that identifying as nonbinary, compared to identifying as transfeminine, was associated with using a lower dose of hormones than prescribed. This gender difference may be related to the specific treatment goals of nonbinary people, which may differ from binary trans people. The lower use of hormones by nonbinary people may suggest a need for prescribers to communicate better with nonbinary patients about their gender-affirmation goals. Notably, there is a paucity of studies on non-binary people’s health in general (Matsuno & Budge, 2017; Scandurra et al., 2019), and no studies to our knowledge have examined the specific motivations and desires of nonbinary people compared to binary trans people in their gender-affirming care. While recent articles have made a case for tailoring hormone regimens to the needs of individual nonbinary patients (D’hoore & T’Sjoen, 2022; T’Sjoen et al., 2019), providers may hesitate to tailor hormone doses due to a lack of expertize in identifying and maintaining tailored regimens. Given recent research suggesting that nonbinary people more frequently have to educate their providers about the needs of trans people than binary trans people (Reisner & Hughto, 2019), it is possible that nonbinary people are not being prescribed hormone doses that meet their gender-affirmation goals and therefore feel compelled to tailor their dosage themselves. Future research into intra-transgender community priorities and concerns around hormone use is important to fully understand gender differences in how trans people take their prescribed hormones. Moreover, as this study did not ascertain specific gender-affirming hormones (e.g., estrogen vs. testosterone), understanding which hormones were being prescribed and used among nonbinary people would be important to understanding and contextualizing how to best address the needs of this group.

Lastly, while our sample did not reflect concordant reporting of taking more and less hormones than prescribed, it is conceptually possible that individuals may take more and less hormones at different times—a potential point for future research investigation. The timing and circumstances of when modified dosing behaviors occur should be explored in future longitudinal, mixed-methods studies (e.g., ecological momentary assessments). Findings from such studies could be used to identify strategies to promote adherence and maintenance of hormone use among trans populations.

### Limitations.

This study has methodological limitations that should be considered when interpreting findings. As a cross-sectional study, causality cannot be determined. Moreover, given the use of convenience sampling and the web-based survey, it is possible that the final sample is not representative of the entire trans population, including those who do not have access to the internet. Additionally, the measures used here are based on self-report, which is subject to bias. Although the racial/ethnic composition of the sample reflected that of the Massachusetts and Rhode Island populations (U.S. Census, 2020a, 2020b), results from this study are not generalizable to all trans people in the US, particularly communities largely comprised of racial/ethnic minority populations. Additionally, although we oversampled communities of color and recognized the heterogeneity of racial/ethnic minority communities, due to the lower percentage of participants from each racial/ethnic minority group, we had to combine all non-White participants as a single group (i.e., people of color). This analytical approach may have masked important differences in hormone usage by race/ethnicity. Ethnoracial identity in the context of healthcare provision and access is best used as a proxy for identifying individuals exposed to systemic racism (Lett et al., 2022). Future studies of adherence should capture a more ethnoracially diverse cohort and include direct measures of systemic racism and discrimination to understand if and how hormone regimen adherence may vary due to the noxious exposure to racism (Lett et al., 2022).

## Conclusions

In sum, the present study found the majority of trans individuals surveyed had used hormones at a lower or higher dose than prescribed. Trans individuals who take hormones at doses different than what was prescribed may choose to modify their dose as a means of achieving their gender-affirmation goals, mitigating the adverse physical side effects of hormones, and enhancing physiological and psychological effects of hormones. Structural and interpersonal barriers to care, including cost, lack of insurance coverage, and discrimination, were also found to be key drivers of taking hormones differently than prescribed. These findings underscore the need to eliminate barriers to taking medically necessary hormones for trans populations and the importance of providers understanding the gender-affirmation goals of their trans patients so that they can prescribe appropriate hormone regimens. Future research should seek to understand how providers determine hormone dosing and communicate safety messages to ensure that trans patients are able to achieve their gender-affirmation goals safely and effectively.

## Figures and Tables

**Table 1:** Sample characteristics of transgender adults reporting hormone use in Massachusetts and Rhode Island (*n* = 379).

	ALL *N* = 379		Took more hormones than prescribed				Took less hormones than prescribed			
			Yes (*n* = 90)		No (*n* = 283)		Yes (*n* = 215)		No (*n* = 161)	
	*n*	%	*n*	%	*n*	%	*n*	%	*n*	%
Demographics										
Young adult										
Yes: (18–29)	193	50.92	51	56.67	137	48.41	106	49.30	85	52.80
No (30+)	186	49.08	39	43.33	146	51.59	109	50.70	76	47.20
Race/ethnicity										
White, non-Hispanic	309	81.96	69	77.53	238	84.40	174	81.31	133	83.12
Person of color										
Total	68	18.04	20	22.47	44	15.60	40	18.69	27	16.88
Asian/Pacific Islander (non-Hispanic)	14	3.71	7	7.87	5	1.77	6	2.80	8	5.00
Black (non-Hispanic)	13	3.45	6	6.74	5	1.77	8	3.74	4	2.50
Middle Eastern/North African (non-Hispanic)	5	1.33	1	1.12	4	1.42	2	0.93	3	1.88
Hispanic/Latinx	10	2.65	2	2.25	8	2.84	7	3.27	3	1.88
Multiple race/ethnicity	26	6.90	4	4.49	22	7.80	17	7.94	9	5.62
Gender spectrum										
Transfeminine	124	32.72	40	44.44	81	28.62	59	27.44	63	39.13
Transmasculine	168	44.33	30	33.33	136	48.06	99	46.05	68	42.24
Nonbinary	87	22.96	20	22.22	66	23.32	57	26.51	30	18.63
Employed for wages										
No	115	30.34	31	34.44	82	28.98	58	26.98	56	34.78
Yes	264	69.66	59	65.56	201	71.02	157	73.02	105	65.22
Low income										
No	173	46.64	45	51.14	127	45.68	91	43.33	82	51.57
Yes	198	53.37	43	48.86	151	24.32	119	56.67	77	48.43
Health care indicators and experiences										
Health insurance covers hormone therapy										
Yes	321	84.70	74	82.22	245	86.57	172	80.00	148	91.93
No/No insurance	58	15.30	16	17.78	38	13.43	43	20.00	13	8.07
Have same provider for primary and gender-affirming care										
No	91	25.63	14	16.87	76	28.25	44	21.89	47	30.72
Yes	264	74.37	69	83.13	193	71.75	157	78.11	106	69.28
Received routine care in the past year										
No	63	17.07	67	76.14	236	85.82	177	83.89	128	82.58
Yes	306	82.93	21	23.86	39	14.18	34	16.11	27	17.42
Received mental health treatment in the past year										
No	255	67.28	33	36.67	86	30.39	60	27.91	61	37.89
Yes	124	32.72	57	63.33	197	69.61	155	72.09	100	62.11
Avoided routine care due to gender-based mistreatment in the past year										
No	284	81.14	64	75.29	218	83.21	163	79.90	120	82.76
Yes	66	18.86	21	24.71	44	16.79	41	20.10	25	17.24
Avoided routine care due to cost in the past year										
No	260	74.50	60	71.43	200	76.05	145	70.73	115	79.86
Yes	89	25.50	24	28.57	63	23.95	60	29.27	29	20.14
Provider refused treatment in the past year										
No	326	93.68	77	91.67	246	94.25	186	92.54	139	95.21
Yes	22	6.32	7	8.33	15	5.75	15	7.46	7	4.79
	Mean	SD	Mean	SD	Mean	SD	Mean	SD	Mean	SD
Number of major gender-based discrimination experiences (continuous)	1.74	1.88	2.40	2.35	1.54	1.66	1.86	2.05	1.64	1.68

**Table 2:** Unadjusted and adjusted lasso-selected logistic regression results modeling hormone medicating behaviors among transgender adults in Massachusetts and Rhode Island (*n* = 379).

	Took more hormones than prescribed						Took less hormones than prescribed					
	Unadjusted OR	95% CI	*p*-value	Adjusted OR	95% CI	*p*-value	Unadjusted OR	95% CI	*p*-value	Adjusted OR	95% CI	*p*-value
Demographics												
Young adult												
Yes: (18–29)	Ref.			Ref.			Ref.			Ref.		
No (30+)	0.71	0.44–1.16	0.170				1.15	0.75–1.74	0.513			
Race/ethnicity												
White, non-Hispanic	Ref.			Ref.			Ref.			Ref.		
Person of color	1.56	0.86–2.83	0.137				1.13	0.67–1.90	0.640			
Gender spectrum												
Transfeminine	Ref.			Ref.			Ref.			Ref.		
Transmasculine	0.44	0.25–0.76	**0.004**	0.45	0.23–0.88	**0.021**	1.55	0.95–2.52	0.070	1.53	0.87–2.69	0.140
Nonbinary	0.61	0.33–1.12	0.114	0.63	0.29–1.38	0.254	2.02	1.17–3.50	**0.011**	2.06	1.05–4.04	**0.035**
Employed for wages												
No	Ref.			Ref.			Ref.			Ref.		
Yes	0.77	0.46–1.30	0.339	0.51	0.24–1.07	0.097	1.44	0.93–2.22	0.095	1.69	0.94–3.04	0.079
Low income												
No	Ref.			Ref.			Ref.			Ref.		
Yes	0.8	0.48–1.32	0.392	0.41	0.20–0.82	**0.012**	1.39	0.90–2.13	0.128	1.94	1.13–3.32	**0.015**
Healthcare experiences and discrimination												
Health insurance covers hormone therapy												
Yes	Ref.			Ref.			Ref.			Ref.		
No/No insurance	1.39	0.74–2.61	0.300				2.84	1.36–5.92	**0.005**	4.27	1.73–10.56	**0.002**
Have same provider for primary and gender-affirming care												
No	Ref.			Ref.			Ref.			Ref.		
Yes	1.94	0.96–3.89	0.062	2.14	1.04–4.44	**0.039**	1.57	0.98–2.53	0.057	1.50	0.85–2.63	0.154
Recieved routine care in the past year												
No	Ref.			Ref.			Ref.			Ref.		
Yes	0.52	0.28–0.96	**0.039**	0.66	0.31–1.36	0.264	1.09	0.63–1.89	0.738			
Received mental health treatment in the past year												
No	Ref.			Ref.			Ref.			Ref.		
Yes	0.75	0.45–1.26	0.284				1.57	1.01–2.43	**0.041**	2.00	1.14–3.48	**0.014**
Avoided routine care due to gender-based mistreatment in the past year												
No	Ref.			Ref.			Ref.			Ref.		
Yes	1.62	0.90–2.92	0.104				1.20	0.67–2.17	0.530			
Avoided routine care due to cost in the past year												
No	Ref.			Ref.			Ref.			Ref.		
Yes	1.26	0.70–2.28	0.426				1.64	0.97–2.76	0.063			
Provider refused treatment in the past year												
No	Ref.			Ref.			Ref.			Ref.		
Yes	1.49	0.54–4.10	0.440				1.60	0.55–4.60	0.382	2.17	0.73–6.43	0.159
Number of major gender-based discrimination experiences (continuous)	1.25	1.10–1.42	**<0.001**	1.25	1.08–1.44	**0.003**	1.06	0.94–1.19	0.293			

Bold values indicate significant findings at *p* < 0.05.

Note: Model included bootstrapping procedure of 1000 iterations.

**Table 3:** Reasons for taking more or less hormones than prescribed.

	*n* ^a^	%
Reasons for taking more hormones (*n* = 90)		
Believe that it is not the right prescribed dose	33	36.7%
Hormones make me feel good	32	35.6%
To speed up transition/gender-affirmation process	24	26.7%
To make up for missed doses^b^	15	16.7%
Provider mistrust	10	11.1%
Suggested by friends	4	4.4%
Reproductive health concerns (menstruation, cramping)^b^	4	4.4%
Imprecise practice of dose administration (sometimes it leaks out of the syringe, sometimes hard to tell where the specific line is)^b^	2	2.2%
Reasons for taking less hormones (*n* = 215)		
Forgot to take it	150	69.8%
Forgot to pick up hormone prescription	59	27.4%
Hormone cost	38	17.7%
Travel barrier to get prescription	28	13.0%
Syringe concerns (e.g., phobia, pain, anxiety)^b^	20	9.3%
Health insurance issues (e.g., lack of, delay in approval, changes in in-network provider)	16	7.4%
Believe that it is not the right prescribed dose	11	5.1%
Unsure about taking hormones	10	4.7%
Worried about weight gain	9	4.2%
Pharmacy issues (e.g., delays/risk of unavailability leads to rationing doses, stigma by pharmacists, refill difficulty)^b^	8	3.7%
Provider mistrust	5	2.3%
Worried about negative side effects with other medicines	4	1.9%
Hormones make me feel sick	3	1.4%
Other physical health concerns (e.g., hair loss, acne issues)^b^	3	1.4%
It’s not always available through friends/online	1	0.5%
Adviced by provider not to take it	1	0.5%
Reliance on others to administer it^b^	1	0.5%

a Check all that apply.

b Emergent themes from write-in data.

## Data Availability

Given that this study contains data with potentially sensitive information, data from this study are available upon request. Contact the The Fenway Institutional Review Board (IRB) Committee (information@fenwayhealth.org) for data requests.
